# Monitoring patient safety in primary care: an exploratory study using in-depth semistructured interviews

**DOI:** 10.1136/bmjopen-2015-008128

**Published:** 2015-09-08

**Authors:** Rajvinder Samra, Alex Bottle, Paul Aylin

**Affiliations:** Dr Foster Unit at Imperial, Department of Primary Care and Public Health, Imperial College London, London, UK

**Keywords:** PRIMARY CARE, QUALITATIVE RESEARCH

## Abstract

**Objectives:**

To explore how information and data are used to monitor patient safety and quality of primary care by professionals working in, or supporting, primary healthcare.

**Design:**

Qualitative study of semistructured interviews with a directed content analysis of transcripts.

**Setting:**

North-West London, UK.

**Participants:**

21 individuals from various levels of the primary healthcare system were recruited, including general practitioners, practice nurses, practice managers, members of Clinical Commissioning Group (CCG) governing bodies, and senior members of regional patient safety teams.

**Results:**

Participants described being overwhelmed with complicated data which lacked any meaningful analyses about safety and quality. There was also a lack of clarity over which patient safety events are expected to be reported or monitored. Participants also reported uncertainty on whose responsibility it was to act on patient safety information or concerns. At the practice level, there was a range of disincentives for responding to and acting on safety issues and concerns, with few reported benefits. Participants made recommendations to improve future monitoring.

**Conclusions:**

There is a need for clearer information in the form of specific guidelines, policies and procedures with regard to who monitors patient safety in primary care, what is monitored and how it should be monitored.

**Strengths and limitations of this study**

- This study employed a multiprofessional participant group at various levels of the primary care system, providing a more realistic account of the complexities of monitoring patient safety in primary care.
- The interview topics were focused on current barriers and facilitators to monitoring patient safety which, combined with the use of a directed content analysis, allowed an in-depth exploration of what works and what does not work for patient safety monitoring in primary care.
- Participants offered detailed and specific recommendations to improving the use of data to monitoring patient safety in primary care.
- These findings may not be generalisable to other healthcare agencies and organisations involved in primary care that were not represented in this study.
- This study took place in North-West London and the results may not reflect the experiences of those working in other areas.

## Introduction

It is estimated that between 37 and 600 patient safety incidents occur in UK primary care per day.^1^ Despite this, the nature and extent of harm in primary care are still not well understood.^1^ ^2^ Beyond the basic reporting and publishing of quality and safety outcome indicator data,^3^ it is also unclear how primary care organisations, such as Clinical Commissioning Groups (CCGs), their member practices and local NHS England area teams, collaborate to monitor patient safety. Past primary care patient safety research often has a tightly focused area of enquiry such as general practice computer systems or E-prescribing,^4–6^ incident reporting^7^ and safety culture.^8^ ^9^ However, primary care is diverse, complex and collaborative,^10^ and a less top-down approach has been recommended.^11^ Additionally, studies that indicate ways to improve patient safety systems^4^ tend to assume that provision of an improved system is sufficient for its uptake, which is not necessarily the case,^6^ ^12^ especially given the unprecedented time and resource demands on UK primary care staff.^13^ ^14^ There exists an opportunity to consult primary care staff for a realistic picture of whether and how they collaborate to monitor patient safety. Drawing on the tradition of qualitative enquiry into patient safety,^4^ ^6^ this study uses informant interviews in North-West London (NWL) to explore how patient safety is currently monitored in primary care settings. Patient safety is defined as the “reduction of risk of unnecessary harm associated with healthcare to an acceptable minimum”.^15^

## Methods

### Study design

This study used in-depth semistructured interviews which were suited to exploratory aims of the study.^16^ An interview guide was used to ensure that some core questions were asked of all participants, but also allowing flexibility to follow-up novel information.^17^

### Participants and procedures

Twenty-one individuals participated in the study. Individuals working in general practitioner (GP) practices and those supporting and monitoring the delivery of these services (CCG governing body members and the NHS England regional patient safety and quality teams) in NWL were eligible for the study. Email invitations were distributed to members of the governing bodies of the eight NWL CCGs. Snowballing was employed through the use of email lists to CCG-member GP practices which allowed for identification and access to further relevant professionals. This method is useful when the sampling frame is unknown and traditional random sampling is implausible.^18^ Once data saturation was reached after 21 interviews,^19^ no further participants were recruited.

### Data collection

Interviews took place between June and September 2014 in a private office at the participants’ workplace. Interviews ranged from 29 to 47 min and were audiorecorded. Interviews were conducted by one member of the research team (RS) who had previous training in interviewing healthcare staff and holds a PhD in applied psychology. Participants were informed that RS was a research associate and did not hold any clinical or management roles in any healthcare organisations. The interview guide was piloted on the first three participants. For all interviews, participants were asked to first provide their own description of patient safety, and then instructed to consider patient safety as relating to when a patient has been harmed or injured as a result of their care or lack of care. An interview guide is included (box 1). This study was a service evaluation^20^ and therefore did not require NHS Research Ethics Committee approval,^21^ but local research governance permissions were sought. Participants were given a study information sheet and their informed consent was obtained.

Box 1 **Interview guide**

1. What does the phrase ‘patient safety’ mean to you?
2. Can you describe any ways of identifying cases where there have been medical errors or patients have been harmed by their care?
3. Are there any ways of sharing information about patient safety events or near misses with others who work in primary care?
  - *Prompts*: Can you describe these?
  - How often does this happen?
4. If there was a growing concern where the same patient safety adverse event was occurring in a particular area/practice/your practice, how would this usually be flagged up to you?
5. Are there any ways in which you think the data supporting patient safety in primary care could be improved?

*Prompt*: Do you think these analyses adequately represent trends in patient safety and quality of care?

- 6. With the information and feedback channels that exist, do you feel that primary care practices where there are safety issues are currently being identified with a good degree of accuracy?

*Prompts*: Why/why not?

How could this be done better?

- 7. In terms of monitoring patient safety in primary care, what makes this difficult for you?
- 8. Are there any things that would make it easier to monitor patient safety in primary care?

### Data analysis

Interview transcripts were subjected to a directed content analysis,^22^ a form of thematic analysis in which some coding categories are predetermined in line with the aims of the study.^23^ ^24^ These predefined categories were: the current methods of identifying patient safety events; perceived barriers and facilitators; and recommendations for the future. Transcripts were coded by one member of the research team (RS). Any other relevant statements were given new codes at this stage, which culminated in the final coding framework. The coded data were investigated for relationships which linked them. These became subthematic-level data and relationships between subthematic data became the overarching main themes. The final thematic framework (box 2) was developed by one researcher.

Box 2 **Thematic framework: monitoring patient safety in primary care**

1. Access to information and data
  - The overwhelming number of performance measures
  - Variability in receiving patient data in the general practitioner surgery
  - Access to (meaningful) analyses/data about safety
2. Clarity of policies and guidelines
  - Operationalisation of patient safety and patient safety-related events
  - Local variation in policies and protocols
3. Responsibility and action
  - Ownership of the issue
  - The lack of visible monitoring in primary care
  - Prioritising other pressures over safety and quality
  - Disincentives to report potentially serious incidents
  - Dependence on informal human vigilance and feedback

## Results

Twenty-one individuals participated in the study (table 1). The three main themes are presented with data from the interview transcripts (with the participant identifier) to reflect the main points of interest.

**Table 1:** Interview participant characteristics

Identifier	Professional role/s	Gender	Job experience (in years)
CCG1	Clinician* with CCG governing body role	Male	24
GP1	GP	Male	1
CCG2	Clinician with CCG governing body role	Male	8
GP2	GP	Female	8
GP3	GP	Male	15
CSU1	Safety and quality executive at NWL CSU	Female	8
CCG3	Clinician with CCG governing body role	Female	18
NHSE1	Safety/quality executive at NHSE	Female	1
GP4	GP	Female	13
NHSE2	Safety/quality executive at NHSE	Female	1
GP5	GP	Female	12
GP6	GP	Female	12
CCG5	Clinician with CCG governing body role	Male	20
CCG6	Clinician with CCG governing body role	Male	20
PM1	PM	Female	16
GP7	GP	Male	25
CCG7	Safety/quality executive in CCG	Male	2
NHSE3	Safety/quality executive at NHSE	Female	1
CCG8	Clinician with CCG governing body role	Female	28
NHSE4	Safety/quality executive at NHSE	Male	2
PM2	PM	Female	6

*Clinicians—job experience denotes years worked after medical qualification; non-clinician—job experience denotes years worked in current role.

†In this study, the term ‘clinician’ (practitioners with patient contact) denotes GPs, nurses or secondary care practitioners—exact profession is not specified as data would be identifiable.

CCG, Clinical Commissioning Group; CUG, Commissioning Support Units; GP, general practitioner; NHSE, NHS England; NWL, North-West London; PM, practice manager.

### Theme 1: access to information and data

Participants reported an overwhelming number of performance measures which did not reflect patient safety but were considered a mechanism for remuneration: “You get fixated on depression because that's what you're being paid for…So you tend to ignore other mental health co-morbidities because depressions the one you're focusing on” (GP1). Individuals working in general practice reported not knowing which harms they should be evaluating to monitor safety: “If they set out really clearly, ‘we believe that these five things would really improve patient safety and so we want you to report to us, every single medication error, every single needle stick injury’…We could then do that. I suppose that's the problem, it's just so wide at the moment in primary care that we're never really sure” (PM2).

GPs simultaneously spoke of too little and too much information on discharge summaries (“Lots and lots of information about various tests that the patients have had but it's information that I actually don't have the expertise to interpret” CCG3). Not knowing what occurs to patients after a referral to district nursing was a concern (“Referral to district nursing—it's like dropping it into a black hole. You don't know if the nurse has ever seen the patient or whether what you've asked to be done has been done” CCG3). There were issues around receiving letters/communication in a format incompatible with the system holding the patient records in the practice, in which case pertinent information was manually entered into the patient's record by practice staff which created a lot of opportunity for error: “If there's ten or fifteen medications which is not uncommon with patients, that could be a really big problem…every possible error, from transcription error on names of medication or dosages, lengths of time that the patient's expected to be on the medication—be it permanent or short-term—loads of room for error on that” (GP7).

Participants from NHS England reported that no core metrics were routinely analysed for safety monitoring (“It's very underdeveloped…The honest answer is we don't have a set of metrics that we look at” NHSE1). Instead, the accessible data were manually scanned for red flags. These data may be discussed at operational group meetings, which take place every 1 or 2 months, but these meetings were described as fixated on trying to get through the information collected through secondary care quality and safety indicators: “We're trying to look at those. There's hundreds. There's literally about two hundred. Three hundred” (CCG7). Participants from management organisations (NHS England, CCG governing boards and the Commissioning Support Units (CSU)) tended to report that safety data (such as serious incidents) and complaints were distributed across and within a number of organisations (“It's distributed across NHS England: the revalidation team, the performance list team, the contract managing team, and so on.”) with recommendations to collate this information in the future.

### Theme 2: clarity of policies and guidelines

Across participants, there was no consensus on the meaning of the term ‘patient safety’ in relation to primary care because the concept was considered vague or they described it as everything in the medical process: “It could mean all sorts of things…So it's everything actually. Patient safety is everything we do” (GP5). Additionally, it was not clear what constituted a serious incident, whether reporting was mandatory and where to report them. Participants explained that serious harm and never events had an acute focus and that general practice was comparatively safer: “You think ‘well, compared to that, our risk is zero’ so it feels like an overreaction to follow some of this process” (PM2). Different methods for reporting patient safety incidents (such as emailing or ringing up a local or national team at NHS England, completing an incident report form from NHS England, or anonymously reporting through the National Reporting and Learning System (NRLS)) appeared to result in confusion about which agency the information was received by and which of these methods satisfied mandatory reporting requirements, even among those at the CCG governing board level: “I struggle when I ask the question to get any sense of the mechanisms by which GPs might report, or anybody in general practice might report, the mechanisms by which patients might report their concerns…I have no idea. And my suspicion would be that nobody has any idea” (CCG6).

Some GPs reported having carried out informal safety monitoring evaluations or audits in the past. This type of monitoring was optional, variable and time-intensive: “Looking at your prescribing rates compared to somebody else…GPs are having to do that by hand and that's why they might do it one year, skip it another year…you might be looking at methotrexate and all the anti-tumour drugs that might be prescribed. So you're covering so many areas you do not have time to do every single one. If somebody could do that and just present the data” (GP2). Multiple GPs mentioned that they needed more guidance about which drugs to monitor and the frequency of medication reviews (especially for long-term medications and high-risk drugs) for repeat prescribing. Specifically, they were interested in: “All the drugs that patients take where monitoring is recognised and recommended and then, what are the monitoring intervals and what are the ranges that are acceptable?” (CCG2).

### Theme 3: responsibility and action

At the management level, there were conflicting responses about whose responsibility it was to monitor patient safety in primary care. CCG governing body members generally reported that monitoring safety was outside of their remit and lay with NHS England, whereas participants from NHS England saw themselves as part of a collaborative effort with CCG governing bodies and the practice networks. There was mention of the fact that the CCGs do “have this vague responsibility for quality [improvement] in general practice, whatever that's supposed to mean” (CCG3). Participants from NHS England and the CCG governing bodies also reported conflicting responses about who monitors patient safety in urgent care centres and for out-of-hours services, with some GPs stating that it appeared that nobody was monitoring these services: “And urgent care centres are making huge amounts of money but the quality of care—who's questioning that?…Do we have any data on the safety of prescribing or drug errors or prescribing errors in urgent care centres, is anyone looking at that—even out of hours?” (GP6).

Participants from CCG governing bodies and NHS England spoke at length about how GP practices managed their incidents locally and made use of networks or peer groups (of 6–11 practices meeting monthly) to check-up on each other and share information. However, participants working in GP practices reported using these meetings to make sense of recent changes to policies instead of discussing patient safety. Additionally, poor performing surgeries may work together with similar others in order to avoid detection, referred to as “collusion” (CCG8). Almost all participants reported that they had local knowledge about which practices were and were not safe and that the focus of patient safety monitoring should be on these types of practices, with some identifying single-handed or two-handed practices as a cause for concern: “There's a pattern of poor performance in men, over 50, who trained abroad, who didn't train in the UK, and who are single handed, small, very small practice and probably have got poor premises. They're high indicators of underperformance…the trouble is the trained abroad stuff, is politically very sensitive” (NHSE4).

Participants who worked in practices frequently described the difficulty of managing time pressures on an average working day. GPs reported not having the time to fill in incident report forms or conduct safety audits: “Although there are areas where we are asked to collect data, we're just so busy and so stretched that we don't really do it…We hold the minor surgery service and in theory we try to run an audit of, or we try to keep a record of if there's post-operative infections. But actually to do that properly, it's really difficult, so we don't do it properly” (CCG2). Many GPs explained that it was difficult to be safe in a 10–15 min appointment in which patients often brought multiple serious health and social concerns to the same appointment due to having access to care issues: “But I think primary care's really dangerous right now to be honest. I am getting quite near to the feeling that I don't want to carry on doing it” (CCG2).

For a number of reasons, the recommended protocol for dealing with a potentially serious incident was not always followed: “So what I should be doing is logging it on that, sending it off to them. To be honest, almost never happens” (CCG3). Other than lack of time, participants feared blame, organisational and personal repercussions that were amplified if the potentially serious incidents involved a senior GP. Multiple GPs reported the belief that NHS England would not or could not act on the evidence, and that this deterred them: because “the onus is on that GP and so on top of your normal workload, and for the fear of being isolated and victimised, who's going to do that? It's easier to walk away from it” (GP6). The failure to report incidents outside of the practice was attributed to a number of factors, including a workplace culture that mistakes were deemed to be “within acceptable limits even though in fact if one was to have the hard evidence and comparative with what's going on on a national basis, you might find that you are a complete outlier” (GP7).

### Summary

Participants’ report of the barriers to monitoring patient safety in primary care are outlined in box 3 and their recommendations for the future are provided in box 4.

Box 3 **Perceived barriers to using data to monitor safety**

Perceived barriers

- Lack of information about district nursing activity delivered to patients’ general practitioner (GP)
- Lack established regional/national protocols regarding monitoring of repeat prescribing or high-risk drugs
- Information received from hospitals is too basic or too complex for GP
- Lack of examples of serious harm or never events that are applicable to primary care settings for which to monitor
- Limitations to involvement of salaried and locum doctors with Quality and Outcomes Framework (QOF) data
- Too many inappropriate pop-up warnings on GP clinical systems
- Limited and unreliable data on serious incidents
- Lack of specifically allocated time to look at practice-held data or QOF statistics on patient safety
- Local practice network meetings designed for some patient safety peer monitoring used for other purposes
- Organisation holding safety data may not have power to investigate patient safety threats
- Majority of time at operational group meetings dedicated to hospital patient safety monitoring
- Lack of safety metrics routinely analysed at NHS England (London region)
- Limited access to existing safety data as they are divided in terms of the organisations that hold them and within departments in these organisations

Box 4 **Recommendations for improving data to monitor safety**

Recommendations

- For hospital information, clearly outline changes to patient medical and/or medication status and clearly outline action plan for general practitioner (GP) follow-up and monitoring
- Share copy of district nursing care plan with GP
- Hospital information should have READ-codes applied to avoid error during information transfer
- Provide data on missed appointments in other parts of healthcare system to patient's GP, especially required for those at high risk (eg, frail, elderly)
- Collate all patient safety and quality information (including complaints) in one source document which is shared within and between organisations that have a duty to monitor patient safety.
- Provide spreadsheet feedback charts (colour coded: red, amber, green) on prescribing rates data relating to safety (eg, non-formulary drugs, drugs with boxed warnings)
- Provide list of 5–10 patient-defined safety events for practices to identify, clinically code in the patient record, and monitor
- Supply rapid discharge summaries from hospital for other serious illnesses (eg, meningitis, sepsis or lower respiratory tract infections)
- Identify all drugs in which monitoring is recognised; provide a list of the recommended monitoring intervals and acceptable ranges
- Need for computerised automatic safety monitoring audits for known risks (eg, unsafe combination of drugs, long-term use of short-term medication)
- Provide a one-page outline on what a patient safety event is, how and where it should be reported for practices to display in waiting rooms
- Provide a safety reporting system for suspected problems which need further investigation
- Provide a safety reporting system which all primary care practice staff have access to
- Up-to-date (live) patient care record shared between all the patients’ NHS healthcare providers

## Discussion

This study's findings demonstrate that patient safety and patient safety events, such as serious incidents, appear ill-defined in primary care, and therefore it is unclear on the ground what is to be monitored. Patient safety monitoring was perceived to be voluntary, with time and resource constraints dictating that other tasks frequently took priority over safety monitoring. At the management level, the information about patient safety was divided between, and within, various organisations. There was an absence of clear and explicit monitoring strategies and ownership of the issue. This study indicates that there may be a need to establish a clear focus on patient safety in primary care, which requires: (1) a detailed operationalisation of core concepts relating to safety in primary care; (2) explicit guidance for the monitoring, detection and reporting of safety concerns is needed for when events fall outside of well-defined acceptable parameters and (3) clear dissemination of this information is needed for all primary care staff (administrative, managerial, clinical, etc) with action points. These findings indicate the need to make the patient safety agenda^25^ more explicit in primary care.

### Strengths and weaknesses

This study incorporated a multiprofessional participant group at different levels of the local healthcare system, resulting in a realistic case study account of the complexities of monitoring patient safety. As a local evaluation study, the involvement of national bodies such as the CQC was outside the remit of the present work. The CQC has recently undergone significant changes to its primary care services team and undertaken a national consultation on monitoring in GP practices which concluded after the closure of this study.^26^ This study used snowball sampling which can result in oversampling similar members of the population;^18^ but attempts were made to access participants in a range of organisations and professional roles. The present study was conducted in a local primary care system and may not be generalisable to areas other than NWL. The coding and thematic framework was developed by one researcher and so offers room for the replication and development of these themes in future work.

### Comparison with other studies

Recent work has identified the failure to define quality in general practice^27^ and, in a similar vein, this study adds that this may extend to *safety* as well as *serious incidents* in primary care. This lack of shared understanding regarding concepts central to safe care stands in stark contrast to creating a National Health Service (NHS) in which safety is front and centre.^25^ Concerns were raised about the failure to monitor out-of-hours care and urgent care centres, which is consistent with reports that monitoring these services has not been a priority for NHS England or the CCGs.^28^ This study found that there does not appear to be a systematic analysis of the vast data set collected on individual practices or a clear sense of who has the responsibility to act on these data at the management levels. Therefore, the findings of this study support general conclusions from past work that there has been a long history of poor analysis of quality and safety data in primary care.^29^ ^30^ This study also supports the growing evidence that the increasing pressures and responsibilities put on GPs and primary care staff are limiting their ability to deal with issues related to patient safety.^13^ ^14^ ^31^

### Implications for clinicians and policymakers

An operationalisation of *patient safety* and *serious incidents* specifically addressing primary care should be explicitly outlined and available in a succinct format. Primary care patient safety reports should be accompanied by clear action points for GP practices. The development and dissemination of brief standardised guidance regarding how, and to whom, serious incidents in primary care are reported is recommended. Participants provided recommendations for improved monitoring of safety, which have clear implications for practice and policy.
